# Assessment of the Efficiency of Combined Seeding Rates of Common Vetch and Ryegrass for Controlling Weed Development in Organic Forage Cultivation Systems

**DOI:** 10.3390/life15050731

**Published:** 2025-04-30

**Authors:** Hüseyin Çağlar, Serap Kizil Aydemir, Koray Kaçan

**Affiliations:** 1 Republic of Türkiye Ministry of Agriculture and Forestry Genotype Registration and Seed Certification Center, 06172 Ankara, Turkey; huseyin.caglar@tarimorman.gov.tr; 2 Department of Field Crops, Faculty of Agricultural and Natural Sciences, Seyh Edebali University, 11230 Bilecik, Turkey; 3 Department of Animal and Rangeland Sciences, Oregon State University, Corvallis, OR 97331, USA; 4 Department of Plant and Animal Production, Ortaca Vocational School, Mugla Sitki Kocman University, 48000 Mugla, Turkey; koraykacan@mu.edu.tr

**Keywords:** common vetch, ryegrass, mixture, dry matter, suppressing weed

## Abstract

Weed suppression is a crucial factor in sustainable agriculture, and optimizing plant mixtures can enhance weed control efficiency. This study evaluates the effects of different mixture ratios of common vetch (*Vicia sativa* L.) and annual ryegrass (*Lolium multiflorum* L.) on forage yield, biomass production, dry matter production, and weed suppression in organic forage cropping systems. Field experiments were conducted during the 2021–2022 growing season at two locations in Turkey: Ankara/Yenikent and Manisa/Beydere, using 11 mixture ratios ranging from 100% vetch to 100% ryegrass. Results showed that ryegrass-dominant mixtures, particularly 10% vetch/90% ryegrass and 30% vetch/70% ryegrass, achieved the highest forage and dry matter yields while maintaining effective weed suppression. Pure ryegrass systems (100% ryegrass) exhibited the highest overall productivity, whereas pure vetch (100% vetch) treatments were less effective in weed control and biomass production. Environmental differences between locations significantly influenced the performance of mixtures, with Manisa/Beydere yielding higher overall results. This study highlights the potential of optimizing vetch–ryegrass mixtures to balance forage yield, weed suppression, and adaptability in organic cropping systems, offering practical insights for sustainable forage production. Ryegrass-dominated mixtures (30% V 70% RG, 60% V 40% RG) have been shown to provide high yields, effective weed suppression, and better nutritional benefits than vetch.

## 1. Introduction

Organic farming aims to enhance environmental sustainability by minimizing the use of chemical fertilizers and pesticides. In this context, organic forage production plays a significant role in achieving economic and environmental goals in livestock farming. However, one of the main challenges in organic farming is weed management. Weeds can negatively impact crop yield and quality, especially in organic systems where the use of chemical herbicides is prohibited. Addressing this challenge requires ensuring sufficient ground cover and utilizing competitive native plant species.

Weeds in organic systems often thrive due to the absence of synthetic herbicides, leading to significant yield losses. According to Gallandt, weed seed banks can persist in organic systems unless actively managed through crop rotations, cover crops, and species competition [1].

Several studies have highlighted the benefits of mixing legumes—particularly vetch species—with grasses like ryegrass. These mixtures improve soil health and suppress weed growth [2,3]. Vetch, by increasing soil nitrogen content, can provide a competitive advantage for other plants, while ryegrass, with its rapid growth and ground-covering ability, can inhibit weed development [4]. However, the effectiveness of these mixtures can be further optimized by using the correct proportions. Additionally, legume–grass intercropping systems have been shown to provide a more stable production structure compared to monocultures, increasing resilience to environmental stress [5].

In Turkey, one of the major challenges in livestock production is the limited availability of high-quality forage. This problem can be mitigated by protecting natural pastures through sustainable grazing and increasing the cultivation of forage crops in field agriculture [6]. As natural pastures continue to decline, the need for high-yield and high-quality forage from agricultural fields becomes increasingly important [7]. Therefore, it is essential to determine the appropriate ratios of legumes and cereals that contain the necessary protein, carbohydrates, and micronutrients for animals [8].

Recent studies suggest that adjusting the planting time and seeding ratios of legume–grass mixtures can yield efficient and sustainable forage. For instance, cultivating these mixtures before summer crops in irrigated areas and rotating them with winter crops in dry regions may offer a strategic solution to seasonal forage shortages [9]. Moreover, cereal–legume mixtures not only balance nutrient profiles but also reduce nutrient leaching and improve water use efficiency [10].

Common vetch (*Vicia sativa*) and ryegrass (*Lolium multiflorum*) are two legumes and grasses, respectively, which have gained attention for their potential in integrated weed management. Common vetch is known for its ability to fix atmospheric nitrogen, thereby improving soil fertility, and providing a competitive edge against weeds [11]. Ryegrass, on the other hand, is recognized for its rapid growth and dense canopy, which can effectively shade out weeds and reduce their establishment [12,13]. The synergy between these two species may enhance weed suppression more effectively than either grown alone. The combination of these two species may offer synergistic benefits, enhancing the overall efficacy of weed suppression in organic systems.

Optimizing their combined seeding rates is crucial for promoting mutual growth and suppressing weed competition in organic systems. The interaction between plant species and their seeding proportions significantly affects weed dynamics. Research by Corre-Hellou et al. showed that biomass allocation and nitrogen capture are strongly influenced by mixture proportions, particularly under low-input conditions [14].

The assessment of combined seeding rates of common vetch and ryegrass is crucial for optimizing their competitive interactions and maximizing their weed-suppressive potential. Research indicates that the interaction between different plant species and their respective seeding rates can significantly influence weed dynamics [15]. By strategically varying the seeding rates of common vetch and ryegrass, it may be possible to achieve an ideal balance that promotes healthy crop growth while simultaneously minimizing weed pressure. Moreover, understanding these ecological interactions contributes to better management practices aligned with organic principles. Incorporating diverse plant species improves biodiversity, soil structure, and resilience to environmental stress. Diversified cropping systems also reduce the risk of pest and disease outbreaks and improve forage quality over time [16].

Furthermore, understanding the ecological interactions between the species can lead to improved management practices that align with organic farming principles. The integration of diverse plant species not only contributes to weed control but also enhances biodiversity, improves soil structure, and promotes resilience against environmental stresses [17]. This study aims to evaluate the efficiency of combined seeding rates of common vetch and ryegrass in controlling weed development within organic forage cultivation systems, providing insights that could inform organic farmers and agronomists seeking to enhance their weed management strategies.

The aim of this study is to determine the most suitable mixture ratios of vetch and ryegrass for organic forage production and to investigate how these mixtures influence weed suppression. While several studies have explored the potential of such mixtures, determining the effects of different ratios will provide more concrete results, especially in terms of local environmental conditions and agricultural practices. Additionally, understanding how these mixtures can enhance the efficiency and sustainability of organic production systems is of great importance.

Therefore, the aim of this study is to determine the most effective mixture ratios of vetch and ryegrass for organic forage production and to assess their weed suppression capabilities under local environmental conditions. This research is expected to provide practical guidance for organic farmers and agronomists and contribute to the development of sustainable weed management strategies.

## 2. Materials and Methods

This study was conducted during the 2021–2022 vegetation period at two locations: Manisa/Beydere and Ankara/Yenikent. Two distinct plant materials were used: Aneto common vetch (*Vicia sativa* L.) and Trinova annual ryegrass (*Lolium multiflorum* L.).

Aneto is a variety developed through the selection breeding method. It has an average plant height of 48.3 cm and a 1000-seed weight of 55.9 g. Trinova, on the other hand, is a tetraploid variety bred through hybridization and selection, with an average plant height of 96.7 cm. The weight of 1000 seeds of Trinova is 2 g.

### 2.1. Climatic Data

**Figure 1:**
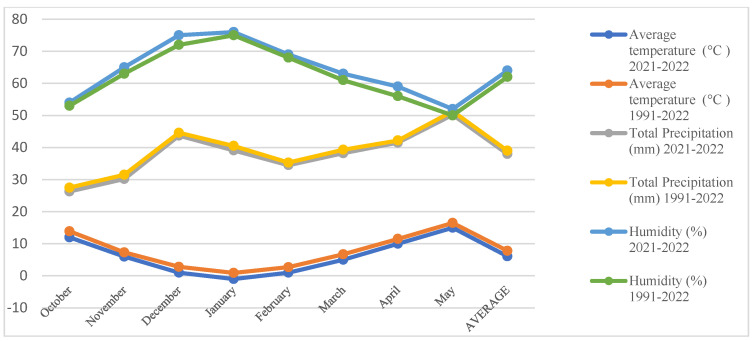
The following data pertains to the climate of the research area for the 2021, 2022 and long-term periods (Ankara/Yenikent).

**Figure 2:**
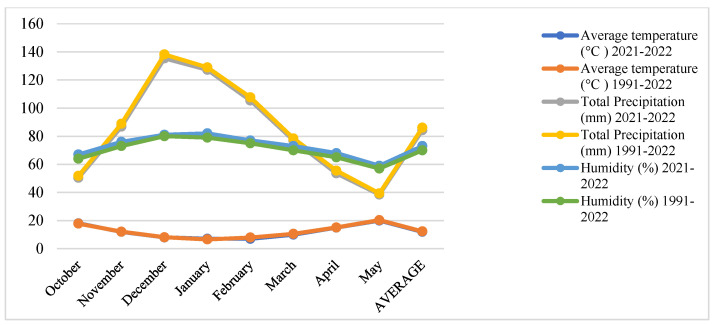
The following data pertains to the climate of the research area for the 2021, 2022 and long-term periods (Manisa/Beydere).

Climatic data for the research area were obtained from the General Directorate of Meteorology and are presented in Figure 1 and Figure 2. Analysis of the data reveals that the long-term average temperature was 7.8 °C in Ankara and 12.3 °C in Manisa, while the average precipitation was 39.0 mm in Ankara and 86.1 mm in Manisa.

### 2.2. Experimental Design and Sowing Procedure

The research was conducted using a randomized block design with three replications at both locations. In the study, 11 different mixture ratios were used. Based on the recommended seeding rates per hectare for common vetch and ryegrass, the seed amounts to be sown per hectare for each mixture were determined, and sowing was carried out accordingly. The seed amounts sown per hectare for each mixture used in the study are presented in Table 1.

Sowing was carried out on 21 October 2021, using a six-row trial seeder with a row spacing of 25 cm.

When examining certain soil properties at a depth of 0–30 cm in the Manisa/Beydere experimental site, the soil was found to be slightly alkaline, non-saline, calcareous, and heavy-textured. In contrast, the soil at the Ankara/Yenikent location was identified as having a brown to gray surface layer, becoming more calcareous and clayey toward the lower horizons.

In line with the technical guidelines provided by TTSM (Turkey Seed Certification and Testing Directorate), 30–40 kg ha of nitrogen (N) and 80–100 kg ha of phosphorus (P₂O₅) fertilizers were applied during sowing. The experimental plot size was 5 m × 1.5 m, equating to a total area of 7.5 m^2^ per plot. Each treatment was replicated three times, resulting in a total of 33 plots. Weed control was performed manually and using a hoe in the spring. 

### 2.3. Measurements and Harvesting

Plant height measurements were made at the full-bloom stage of the vetch. The height of the plants was measured as the distance from the soil surface to the plant tip. In particular, when measuring the height of the vetch, measurements were made by lifting them up by hand to obtain the correct vertical height.

Measurements were made on 10 randomly selected plants for each pure stand and 10 plants per species for the mixtures in each plot. Measurements were taken using a ruler with millimeter graduations and average values were calculated [18].

Harvest timing was determined based on the growth stage of common vetch, specifically when the lower pods were fully formed, and seeds had reached maturity. To minimize edge effects, the two outermost rows of each six-row plot and the first 50 cm from the plot’s edge were excluded from data collection. The remaining 4 m^2^ area was harvested using a mower, and the green forage yield was weighed. The recorded values were then converted to yield per hectare following TTSM guidelines.

From the green forage obtained in each plot, a 500-g sample was randomly selected and dried in a drying oven at 70 °C for 48 h until a constant weight was achieved. Afterward, the sample was left for 24 h to stabilize and then reweighed to determine the dry forage weight. The hay yield per hectare was calculated by multiplying the dry matter ratio by the green forage yield [18].

**Figure 3:**
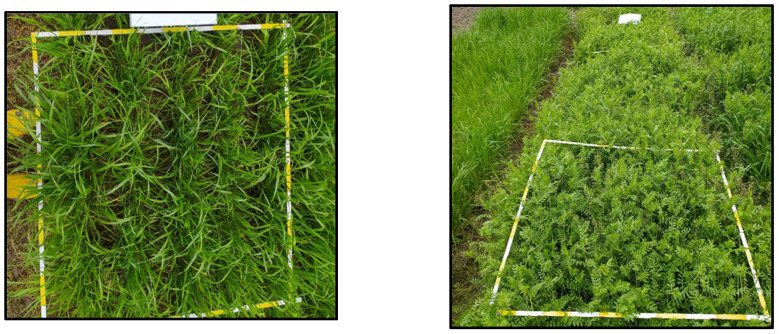
Method for determining weeds in mixed planting systems.

Weed measurements were taken using two 1 square meter frames, placed at random in each plot. Weeds in the frames were collected by hand, their species identified, and the green weight of biomass measured (Figure 3).

The collected weed samples were then dried in a drying cabinet at 70 °C for 48 h until a constant weight was achieved. Subsequently, the samples were equilibrated for 24 h and weighed again to determine the dry biomass weight. The ratio of dry biomass to fresh biomass was calculated and used to estimate the hay yield per hectare.

### 2.4. Statistical Analysis

The data obtained from the study were analyzed for variance using the randomized block design within the MSTAT-C software (2.1) package. The Duncan multiple range test was applied to identify statistically significant differences among the means.

## 3. Results

### 3.1. Plant Height (cm)

The plant height of common vetch was significantly influenced by the planting systems in both locations and the general average (Table 2). The highest plant height was observed under the 100% V planting system across all conditions, with the values being 55.3 cm in Ankara/Yenikent, 75.0 cm in Manisa/Beydere, and 65.2 cm as the general average.

Planting systems with higher proportions of ryegrass (RG) tended to reduce the plant height slightly, with the lowest value recorded at 10% V 90% RG (63.2 cm for the general average). Significant differences were noted among treatments, with lower LSD values indicating reliable distinctions in plant height between groups.

The low coefficient of variation (CV%) across locations and the general average suggests high consistency and reliability in the data. Overall, the results indicate that higher vetch proportions in the mixture support greater plant height, likely due to reduced competition from ryegrass.

The plant height of ryegrass was also significantly influenced by the planting system (Table 3). The pure ryegrass system (100% RG) resulted in the highest general average height (75.6 cm), followed by the 10% V 90% RG system (74.8 cm). As the proportion of vetch increased, the height of ryegrass tended to decrease, with the lowest value (70.3 cm) observed in the 90% V 10% RG system.

The differences in plant height across treatments were statistically significant, with low CV% values indicating consistent results. This suggests that ryegrass performs optimally in pure stands or when present in high proportions in mixed systems. These findings are crucial for understanding the interactions between ryegrass and vetch in mixed cropping systems. The results can guide the determination of optimal mixture ratios for achieving both high-quality forage production and biodiversity in pastures.

### 3.2. Green Forage Yield (kg ha)

The Table 4 presents the average green forage yield values (kg ha) for common vetch and annual grass mixtures in the 2021–2022 growing season under different planting systems. The yields are analyzed based on various mixture ratios and locations (Ankara/Yenikent and Manisa/Beydere). Additionally, the general average yield and success ranking for each planting system are provided (Table 4). The ratio of 100% RG provides the highest yield in both locations. The average yield in Ankara/Yenikent is 24,933 kg ha, and in Manisa/Beydere it is 29,200 kg ha, resulting in an overall average of 27,067 kg ha. This system ranks first in the success order. The 40% V 60% RG mixture results in the lowest yield, with 13,767 kg ha in Ankara/Yenikent and 17,911 kg ha in Manisa/Beydere, giving an overall yield of 15,839 kg ha. This system ranks 11th. The 90% V 10% RG mixture (12,900 kg ha and 33,244 kg ha, respectively) ranks second, with a particularly high yield in Manisa/Beydere. Other mixtures, particularly those with 50% and 60% RG, also show good performance. For example, the 60% V 40% RG and 50% V 50% RG mixtures resulted in average yields of 21,417 kg ha and 19,717 kg ha, respectively. The F and CV (coefficient of variation) values are important for understanding the variability and reliability of the data. Especially at extreme mixture ratios like 100% RG and 100% V, there are significant differences in yields. This indicates that different mixture ratios have an impact on yield, and the data is relatively reliable. The data reveals the impact of different mixture ratios of vetch and grass on green forage yield. Based on the results, the 100% RG mixture provided the highest yield, while the 40% V 60% RG mixture gave the lowest yield. However, the yields of different mixtures vary depending on the planting region and climatic conditions. In general, mixtures with a higher proportion of (100% RG) tend to result in higher yields.

These findings can be useful for developing agricultural strategies to select more efficient and environmentally adaptable plant mixtures.

### 3.3. Dry Matter Yield (kg ha)

Observations on the dry hay yield of common vetch and rye grass were conducted separately for the Ankara and Manisa locations. The results of the dry matter yield values obtained are presented in Table 5. In addition, when the average dry matter yield values obtained from common vetch and Ryegrass plants are examined. The highest dry matter yield in Ankara/Yenikent was observed in the 10% V 90% RG mixture (4512 kg ha), whereas in Manisa/Beydere, the 90% V 10% RG mixture yielded the highest dry matter (6505 kg ha). The differences between the locations are due to environmental and climate differences. It is thought that the high yield in Manisa/Beydere location in particular is due to the high amount of rainfall in November and December.

The general average indicates the 100% RG system as the most successful in overall dry matter yield (4780 kg ha), followed by the 90% V 10% RG mixture (4673 kg ha). Systems with a higher percentage of Ryegrass (RG) generally performed better in both locations, suggesting its strong contribution to biomass production. However, high variability (as reflected by high CV%) implies that yield stability across regions may vary depending on environmental factors. Significant differences among the treatments (indicated by F values) confirm that planting mixtures influence dry matter yield. The LSD values highlight the minimum difference needed to declare significant differences between the means. Mixtures with higher proportions of RG (e.g., 100% RG, 90% V 10% RG, and 30% V 70% RG) rank highest in terms of average yield. Farmers aiming for high dry matter yields might prioritize mixtures with a dominant proportion of ryegrass, especially in environments similar to the study regions.

### 3.4. The Effects of Treatments in the Experiment on Weeds and Weed Measurement Evaluations (g m^2^)

The 100% vetch (V) treatment resulted in the highest weed fresh (107.0 g m^2^) and dry weight (29.8 g m^2^), indicating poor weed suppression. In contrast, mixtures with higher proportions of ryegrass (RG), particularly 70% V 30% RG and 60% V 40% RG, demonstrated significantly better weed suppression, with fresh weights of 13.7 g m^2^ and 26.0 g m^2^, respectively, and dry weights below 10 g m^2^. Mixtures containing a greater proportion of ryegrass effectively suppressed weeds due to its dense canopy and rapid growth characteristics (Table 6).

The 70% V 30% RG treatment exhibited the best weed suppression, suggesting this mixture is ideal for minimizing weed biomass.

The significant F values and low LSD values confirm that the differences in weed suppression among treatments are statistically valid and not due to random variation. Incorporating ryegrass into vetch-dominated mixtures can effectively reduce weed biomass.

In the experiment conducted in Ankara, the 90% V/10% RG treatment resulted in the highest weed weights, with fresh weights at 71.3 g m^2^ and dry weights at 40.5 g m^2^, but it was the least effective at suppressing weeds. Conversely, the 60% V/40% RG application achieved the best weed suppression, resulting in the lowest green and dry weed weights. Ryegrass (RG) mixtures showed average weed suppression rates of 32.7%, 35.2%, 61.7%, 42.6% and 36.3% in weed green and dry weights, especially those containing 20%, 30%, 40%, 50% and 60% RG, respectively (Table 7).

Mixtures with a higher ryegrass content effectively suppressed weeds thanks to their dense canopy and rapid growth. However, weed suppression rates did not increase in parallel in cropping systems with more than 60% ryegrass.

The significant F values and low LSD values indicate that the differences in weed suppression between treatments are statistically significant and not due to random variation. Incorporating ryegrass into vetch-dominated mixtures can effectively lower weed biomass.

The results from the Ankara/Yenikent experiment were consistent with those from Manisa/Beydere. Analyzing the data in terms of feed values and nutritional quality suggests that ryegrass-containing mixtures should be integrated into planting systems based on the local climate and growing conditions.

### 3.5. The Effects of Common Vetch and Ryegrass Mixture Ratios on Weed Species

*Atriplex patula* (18.40 pieces m^2^) was found to be the densest weed in the trial area in Manisa province. The least common weed species was *Portulaca oleracea* (1.25 weed) and the most common weed was *Amaranthus retroflexus*. The average number of perennial weeds was 16.14, while the total number of annual weeds was 85.37. The highest number of weeds was determined in 90% V/10% RG (20.91 pieces) mixture and the lowest number of weeds was determined in 60% V/40% RG (4.21 weed) mixture (Table 8).

In the Ankara trial area, the most common weed species was *Lolium temulentum* (13.47) and the lowest number was *Silybum marianum* (0.42). The least common weed species were *Silybum marianum* and Tribulus terrestris. The mean number of perennial weeds was 31.32, while the total number of annual weeds was 29.99. The highest number of weeds was recorded in the 90% V/10% RG mixture (12.91 weed), while the lowest number of weeds was recorded in the 10% V/90% RG mixture (1.84 weed) (Table 9).

## 4. Discussion

In this study, the effects of different vetch and ryegrass ratios on the potential of suppressing weeds in organic forage crop production were investigated. The results indicate that mixture ratios significantly affect weed suppression capacity. Similarly, the literature reports that legume–grass mixtures suppress weeds through synergistic interactions [19,20]. 

Our study found that mixtures with a high proportion of ryegrass were more effective in weed suppression. The most effective were 70%V/30% RG, 60%V/40% RG, 50%V/50% RG and others, respectively. Notably, mixtures containing 75% ryegrass exhibited lower weed biomass compared to pure vetch. Similarly, a study by Dhima et al. reported that mixtures with a high proportion of ryegrass were more successful in weed suppression [21].

However, as the proportion of vetch increased, soil nitrogen content also rose [22].This finding is consistent with the ability of legumes to fix atmospheric nitrogen [22]. Nevertheless, the reduced weed suppression efficiency in mixtures with high vetch proportions can be explained by the slower development of vetch and its delayed ground coverage [23].

Additionally, increasing plant diversity is known to contribute positively to ecosystem services [24]. Mixed cropping systems have been reported to provide more stable production and improve soil health compared to monocultures [25]. In this context, the vetch–ryegrass mixtures used in our study demonstrate potential both for weed suppression and for enhancing soil fertility.

In conclusion, while ryegrass has a high weed suppression capacity, an optimal balance should be achieved considering the nitrogen-enhancing effect of vetch. Future studies should explore how this balance can be optimized through different sowing times and management strategies.

The results of this study align with the literature findings on the effects of vetch and ryegrass ratios on weed suppression capacity. Mixtures with a high proportion of ryegrass were observed to be more successful in weed suppression, which can be attributed to the rapid growth ability and dense root system of ryegrass [26]. Similarly, Dhima et al. reported that high ryegrass content mixtures were more effective in suppressing weeds [21].

On the other hand, an increase in the proportion of vetch provides an advantage for long-term productivity by increasing soil nitrogen content. It is well known that legumes enhance soil nutrient value by fixing atmospheric nitrogen [22]. However, the slow growth of vetch and its delayed ground coverage can be limiting factors for weed control [23].

Furthermore, increasing plant diversity positively contributes to ecosystem services and supports sustainable agriculture [24]. Mixed cropping systems offer more stable production and soil health advantages compared to monocultures [25]. (In this context, vetch–ryegrass mixtures appear to have significant potential for both weed suppression and soil fertility enhancement.

## 5. Conclusions

The findings highlight the importance of optimizing vetch and ryegrass mixture ratios for organic forage production. Pure ryegrass systems (100% RG) were the most productive in terms of forage and dry matter yields but offered less diversity in nutrient composition. The variations detected between two diverse geographical territories are presumed to result from climatic and geographical discrepancies.

Key conclusions from this study include:

- Yield performance: Pure ryegrass (100% RG) achieved the highest forage and dry matter yields but lacked nutritional diversity.
- Balanced approach: Ryegrass-dominant mixtures (e.g., 30% V 70% RG and %60 V %40 RG) provided high yields, effective weed suppression, and improved nutritional benefits from vetch.
- Weed control: In regions with high weed pressure, ryegrass-dominant mixtures are recommended due to their superior weed suppression.
- Sustainability and efficiency: Incorporating legumes and grasses in appropriate proportions enhances the sustainability and efficiency of organic forage cropping systems.
- Practical recommendations: Farmers should tailor mixture ratios based on regional and climatic conditions and specific production goals to optimize both yield and weed management.

These findings provide practical guidance for selecting optimal forage mixtures in organic cropping systems.

## Figures and Tables

**Table 1:** The 11 different mixture ratios used in the study and the amount of seeds per hectare in the mixtures.

No	Planting Systems	Vetch (kg ha)	Ryegrass (kg ha)	Total (kg ha)
1	100% RG (Trinova)	0	30	30
2	100% V (Aneto)	100	0	100
3	10% V/90% RG	10	27	37
4	20% V/80% RG	20	24	44
5	30% V/70% RG	30	21	51
6	40% V/60% RG	40	18	58
7	50% V/50% RG	50	15	65
8	60% V/40% RG	60	12	72
9	70% V/30% RG	70	9	79
10	80% V/20% RG	80	6	86
11	90% V/10% RG	90	3	93

**Table 2:** Plant height of common vetch (*Vicia sativa*) grown under different proportions of pure and mixed planting systems (cm).

Planting Systems	Ankara/Yenikent	Manisa/Beydere	General Average
%100 V	55.3 a	75.0 a	65.2 a
%90 V/%10 RG	54.3 abc	74.7 ab	64.5 ab
%80 V/%20 RG	55.0 a	73.7 bcd	64.3 ab
%70 V/%30 RG	52.7 c	74.7 ab	63.7 bcd
%60 V/%40 RG	53.7 abc	73.7 bcd	63.7 bcd
%50 V/%50 RG	54.7 ab	73.7 bcd	64.2 bc
%40 V/%60 RG	53.0 bc	73.7 bcd	63.3 cd
%30 V/%70 RG	53.0 bc	74.3 abc	63.7 bcd
%20 V/%80 RG	54.0 abc	73.3 cd	63.7 bcd
%10 V/%90 RG	53.7 abc	72.7 d	63.2 d
F	*	**	**
CV (%)	1.8	0.9	1.3
LSD	1.69	1.1	1.0

*: Statistically significant at *p* ≤ 0.05. **: Statistically significant at *p* ≤ 0.01. Different letters (a, b, c, etc.) within the same column indicate statistically significant differences among treatments (LSD test, *p* ≤ 0.05). Values sharing the same letter are not significantly different from each other.

**Table 3:** Plant height of ryegrass (*Lolium multiflorum*) under different proportions of pure and mixed planting systems (cm).

Planting Systems	Ankara/Yenikent	Manisa/Beydere	General Average
%100 RG	58.9 a	92.3 a	75.6 a
%90 V/%10 RG	51.9 e	88.7 b	70.3 e
%80 V/%20 RG	54.0 d	88.5 bc	71.3 cde
%70 V/%30 RG	54.8 d	86.2 d	70.5 de
%60 V/%40 RG	55.2 d	87.6 bcd	71.4 bcd
%50 V/%50 RG	55.8 bc	87.1 cd	71.5 bcd
%40 V/%60 RG	54.9 cd	88.2 bc	71.6 bc
%30 V/%70 RG	55.4 bcd	88.9 b	72.2 bc
%20 V/%80 RG	56.5 b	88.2 bc	72.4 b
%10 V/%90 RG	58.3 a	91.2 a	74.8 a
F	**	**	**
CV (%)	1.5	1.0	1.2
LSD	1.45	1.59	1.01

**: Statistically significant at *p* ≤ 0.01. Different letters (a, b, c, etc.) within the same column indicate statistically significant differences among treatments (LSD test, *p* ≤ 0.05). Values sharing the same letter are not significantly different from each other.

**Table 4:** Average green forage yield values (kg ha) of common vetch and rye grass mixtures for the 2021–2022 growing season.

Planting Systems	Ankara/Yenikent	Manisa/Beydere	General Average	Order Success
%100 RG	24,933 a	29,200 b	27,067 a	1
%100 V	14,400 ef	23,200 cd	18,800 d	7
%90 V/%10 RG	12,900 f	33,244 a	23,072 b	2
%80 V/%20 RG	14,333 ef	22,355 d	18,344 d	9
%70 V/%30 RG	17,267 cd	20,844 de	19,056 d	6
%60 V/%40 RG	16,433 de	26,400 bc	21,417 bc	4
%50 V/%50 RG	15,300 def	24,133 cd	19,717 cd	5
%40 V/%60 RG	13,767 ef	17,911 e	15,839 e	11
%30 V/%70 RG	21,200 b	23,466 cd	22,333 b	3
%20 V/%80 RG	17,433 cd	18,667 e	18,050 de	10
%10 V/%90 RG	19,333 bc	17,867 e	18,600 d	8
F	**	**	**	
CV (%)	96.3	92.2	94.4	
LSD	2774.1	364.6.5	2220.1	

**: Statistically significant at *p* ≤ 0.01. Different letters (a, b, c, etc.) within the same column indicate statistically significant differences among treatments (LSD test, *p* ≤ 0.05). Values sharing the same letter are not significantly different from each other.

**Table 5:** Average dry matter yield values (kg ha) of common vetch and annual ryegrass mixtures for 2021–2022 growing season.

Planting Systems	Ankara/Yenikent	Manisa/Beydere	General Average	Order Success
%100 RG	4059 ab	5501 b	4780 a	1
%100 V	2748 fe	4421 cde	3584 fg	9
%90 V/%10 RG	2840 def	6505 a	4673 ab	2
%80 V/%20 RG	2659 f	3679 e	3169 g	11
%70 V/%30 RG	3282 dc	3849 e	3566 fg	10
%60 V/%40 RG	3206 cde	5161 bc	4183 cd	5
%50 V/%50 RG	3252 cd	4880 bcd	4066 de	7
%40 V/%60 RG	3660 bc	3832 e	3747 ef	8
%30 V/%70 RG	3920 b	5274 b	4597 abc	3
%20 V/%80 RG	3902 b	4246 de	4074 de	6
%10 V/%90 RG	4512 a	4142 de	4327 bcd	4
F	**	**	**	
CV (%)	82.3	95.0	91.0	
LSD	485.1	754.2	444.1	

**: Statistically significant at *p* ≤ 0.01. Different letters (a, b, c, etc.) within the same column indicate statistically significant differences among treatments (LSD test, *p* ≤ 0.05). Values sharing the same letter are not significantly different from each other.

**Table 6:** Weed suppression results (fresh and dry weight) of common vetch and annual ryegrass mixtures for 2021–2022 (g m^2^) (Manisa/Beydere).

Planting Systems	Fresh Weight (g m^2^)	Dry Weight (g m^2^)
%100 RG	39.7 e	14.7 de
%100 V	107.0a	29.8 a
%90 V/%10 RG	42.8 e	13.0 e
%80 V/%20 RG	24.0 f	10.7 f
%70 V/%30 RG	13.7 g	9.0 f
%60 V/%40 RG	26.0 f	9.8 f
%50 V/%50 RG	38.0 e	13.3 de
%40 V/%60 RG	53.0 d	15.2 d
%30 V/%70 RG	71.0 c	19.5 c
%20 V/%80 RG	96.7 b	27.0 b
%10 YF/%90 RG	55.5 g	20.8 c
F	**	**
CV (%)	9.4	6.8
LSD	8.3	1.9

**: Statistically significant at *p* ≤ 0.01. Different letters (a, b, c, etc.) within the same column indicate statistically significant differences among treatments (LSD test, *p* ≤ 0.05). Values sharing the same letter are not significantly different from each other.

**Table 7:** Weed suppression results (fresh and dry weight) of common vetch and annual ryegrass mixtures for 2021–2022 (g m^2^) (Ankara/Yenikent).

Planting Systems	Fresh Weight (g m^2^)	Dry Weight (g m^2^)
%100 RG	49.7 bc	22.2 bcd
%100 V	67.7 a	29.3 ab
%90 V/%10 RG	71.3 a	40.5 a
%80 V/%20 RG	48.0 bc	19.2 de
%70 V/%30 RG	46.2 bc	20.3 cde
%60 V/%40 RG	27.3 c	11.7 e
%50 V/%50 RG	40.9 c	25.4 bcd
%40 V/%60 RG	45.4 bc	22.8 bcd
%30 V/%70 RG	52.4 b	25.7 bcd
%20 V/%80 RG	55.1 b	30.0 b
%10 YF/%90 RG	48.4 bc	20.2 cde
F	**	**
CV (%)	11.3	23.34
LSD	9.6	6.4

**: Statistically significant at *p* ≤ 0.01. Different letters (a, b, c, etc.) within the same column indicate statistically significant differences among treatments (LSD test, *p* ≤ 0.05). Values sharing the same letter are not significantly different from each other.

**Table 8:** Numbers of weed species in common vetch and annual ryegrass mixtures for 2021–2022 (weed m^2^) (Manisa/Beydere).

Planting Systems	AMARE	ATXPA	AVEFA	BROIN	CHRSE	CONAR	CYNDA	CYPRO	HORMU	LACSE	LOLTE	MALNE	MATCH	MEDSA	PHRCO	POROL	PAPRH	SETVI	STEME	SONAR	SOLNI	SORHA	TRBTE
%100 RG	-	-	0.42	-	-	0.84	0.84	-	1.25	-	1.25	-	1.00	-	-	-	-	-	-	0.42	-	-	-
%100 V	1.63	1.25	0.84	0.42	-	0.24	-	-	-	-	-	-	0.88	0.42	-	-	-	-	0.42	1.67	-	1.24	2.22
%90 V/ %10 RG	1.18	4.60	0.84	1.67	0.42	0.42	1.25	0.42	0.42	0.42	1.25	0.84	1.25	1.25	-	-	0.42	-	0.84	-	1.25	0.90	1.27
%80 V/ %20 RG	0.95	0.84	1.25	0.84	0.42	1.25	0.42	0.84	-	0.84	-	-	-	0.84	-	-	-	0.42	0.42	1.25	-	-	0.95
%70 V/ %30 RG	1.33	2.93	0.55	-	0.84	0.84	-	1.25	0.84	-	2.09	-	0.42	-	2.51	1.25	-	0.42	-	-	1.25	-	-
%60 V/ %40 RG	0.95	-	-	-	0.75	1.25	0.42	-	0.42	-	-	-	-		-	-	0.42	-	-	-	-	-	-
%50 V/ %50 RG	0.42	-	-	2.09	-	0.84	-	-	0.42	0.33	-	0.42	-	-	-	-	-	-	0.25	-	-	-	-
%40 V/ %60 RG	0.49	1.67	1.25	-	-	-	-	-	-	0.24	0.42	0.42	-	-	-	-	0.84	0.42	-	-	-	0.20	
%30 V/ %70 RG	0.34	1.25	1.47	-	0.42	0.84	-	-	1.25	-	-	0.84	0.22	0.25	-	-	0.42	-		-	-	-	-
%20 V/ %80 RG	0.57	2.93	-	0.84	-	-	0.42	-	-		-				2.09	-	-	-		-	-	-	--
%10 V/ %90 RG	0.27	2.93	0.42	0.84	0.25	-	-		-	-	0.67	-	-	-	-	-	0.42	0.42	-	-	-	-	-

AMARE: *Amaranthus retroflexus*, ATXPA: *Atriplex patula*, AVEFA: *Avena fatua*, BROIN: *Bromus inermiş*, CHRSE: *Chrizentemum segetum*, CONAR: *Convolvulus arvensis*, CYNDA: *Cynodon dactylon*, CYPRO: *Cyperus roduntus*, HORMU: *Hordeum murinum*, LACSE: *Lactuca seriola*, LOLTE: *Lolium temulentum*, MALNE: *Malva neglecta*, MATCH: *Matricaria chamomilla*, MEDSA: *Medicago sativa*, PHRCO: *Phragmites comminis*, POROL: *Portulaca olerecea*, PAPRH: *Raphanis raphanistrum*, SETVI: *Seteria viridis*, STEME: *Stelaria media*, SONAR: *Sonchus arvensis*, SOLNI: *Solanum nigrum*, SORHA: *Sorghum halapense*, TRBTE: *Tribulus terrestris*.

**Table 9:** Weed species in common vetch and annual ryegrass mixtures for 2021–2022 (weed m^2^) (Ankara/Yenikent).

Planting Systems	AMARE	BROIN	CONAR	CYNDA	FUMOF	LOLTE	MALNE	PHRCO	POROL	SETVI	SILMA	SINAR	STEME	SONAR	SOLNI	SORHA	VERHE	TRBTE
%100 RG	0.84	-	-	0.84	-	0.63	-	-	-	1.68	-	-	-	-	-	-	-	-
%100 V	0.42	0.42	2.10	-	0.42	1.05	1.26	-	0.74	-	-	0.84	0.53	0.75	1.68	0.84	-	-
%90 V/ %10 RG	0.32	-	1.26	-	1.26	1.37	0.42	-	0.42	0.90	-	0.42	2.15	1.32	-	-	1.68	1.39
%80 V/ %20 RG	-	-	0.42	-	-	1.47	-	-	-	-	-	0.32	-	0.46	-	-	0.42	-
%70 V/ %30 RG	-	0.46	-	-	1.26	1.58	-	-	-	-	-	-	0.19	0.34	0.42	0.42	-	-
%60 V/ %40 RG	0.15	-	2.10	-	0.84	0.95	-	0.84	-	0.32	-	-	-	-	0.42	0.42	-	-
%50 V/ %50 RG	-	-	0.42	-	-	0.84	-	-	-	-	-	0.11	0.25	0.32	1.68	-	-	-
%40 V/ %60 RG	-	0.32	-	2.65	-	1.79	-	-	-	-	-	-	-	0.32	-	0.42	-	-
%30 V/ %70 RG	-	0.32	-	1.13	-	1.16	-	0.42	-	0.53	0.42	-	-	-	-	0.84	-	-
%20 V/ %80 RG	-	-	-	1.89	-	1.89	-	-	-	-	-	-	-	-	-	-	-	-
%10 Y/ %90 RG	-	-	-	-	-	0.74	-	0.42	-	-	-	-	-	-	0.26	0.42	-	-

AMARE: *Amaranthus retroflexus*, BROIN: *Bromus inermiş*, CONAR: *Convolvulus arvensis*, CYNDA: *Cynodon dactylon*, FUMOF: *Fumaria officinalis*, LOLTE: *Lolium temulentum*, MALNE: *Malva neglecta*, PHRCO: *Phragmites comminis*, POROL: *Portulaca olerecea*, SETVI: *Seteria viridis*, SILMA: *Silybum marianum*, SINAR: *Sinapis arvensis*, STEME: *Stelaria media*, SONAR: *Sonchus arvensis*, SOLNI: *Solanum nigrum*, SORHA: *Sorghum halapense*, VERHE: *Veronica hederifolia*, TRBTE: *Tribulus terrestris*.

## Data Availability

The data presented in this study are available within the article.
